# Reviewed article: Moraes LP, Raposo LM. Impact of Vaccination and SARS-CoV-2 Variants on Severe COVID-19 Outcomes: A Cross-Sectional Study, Brazil, 2021-2022. Epidemiol Serv Saude. 2025:34;e20240613

**DOI:** 10.1590/S2237-96222025v34e20240613.b

**Published:** 2025-09-01

**Authors:** Michelle Fernanda Borges da Silva

**Affiliations:** 1 Fiocruz, Rio de Janeiro, RJ, Brazil Fiocruz Rio de Janeiro RJ Brazil

## Peer review B

## Questionnaire

Does the manuscript contain new and significant information to justify publication? Yes

Does the Abstract (Summary) clearly and accurately describe the content of the article? Yes

Is the problem significant and concisely stated? Not applicable

Are the methods described comprehensively? Yes

Are the interpretations and conclusions justified by the results? Yes

Is adequate reference made to other work in the field? Yes

## Manuscript Structure

Length of article is: Adequate

Number of tables is: Adequate

Number of figures is: Adequate

**Please state any conflict(s) of interest that you have in relation to the review of this paper (state “none” if this is not applicable).**

None

**Interest:** Good

**Quality**: Good

**Originality:** Below Average 

**Overall:** Good

**Recommendation:** Minor Revision

## Comments

Prezados autores, Escrevo para apresentar minha avaliação do artigo intitulado "Impacto da vacinação e variantes do SARS-CoV-2 nos desfechos graves da COVID-19 no Brasil, 2021-2022: estudo transversal" (RESS-2024-0613). A temática abordada é de grande relevância e contribui significativamente para a compreensão da dinâmica da COVID-19 no contexto brasileiro. A revisão da literatura é abrangente e a metodologia empregada é adequada para responder às questões de pesquisa propostas. No entanto, identifiquei alguns pontos que poderiam fortalecer ainda mais o estudo. Classificação dos vacinados: Sugiro uma revisão da classificação dos indivíduos como vacinados e não vacinados na Tabela 2. A inclusão dos "parcialmente vacinados" na categoria de não vacinados merece uma análise mais aprofundada, considerando o potencial impacto dessa classificação nos resultados. Termo "indivíduos mais velhos": Recomendo uma maior precisão na utilização do termo "indivíduos mais velhos". A definição de faixas etárias específicas permitiria uma análise mais detalhada da heterogeneidade da resposta vacinal entre os diferentes grupos etários. Duração da imunidade: A discussão sobre a duração da imunidade induzida pela vacina, especialmente em idosos, é fundamental para a compreensão dos resultados e traz uma discussão atual sobre a duração da imunidade e necessidade de doses atualizadas.

Acredito que a inclusão dessas considerações enriquecerá a discussão e fortalecerá as conclusões do estudo. 

Geral: rever termos “indivíduo mais velho” e “idoso”

Resumo: Sem considerações

Introdução:

1. No parágrafo: “Até setembro de 2024, o Brasil já havia administrado mais de 520 milhões de doses de vacinas monovalentes (6). A introdução das vacinas bivalentes fortaleceu a resposta imunológica contra a ômicron e suas subvariantes, com mais de 35 milhões de doses aplicadas, destacando a importância da imunização contínua no controle da pandemia (6).” , incluir percentual da população vacinada nos períodos descritos.

Método:

2. Na subseção “Contexto”, os autores descrevem de forma suscinta a base de dados utilizada, sugere-se ampliar a informação da base de dados para que o leitor tenha a dimensão no contexto nacional. 

3. Na linha 36: “O período de estudo coincide com a campanha de vacinação contra a covid-19”, seria com o “início”, sugere-se reescrever.

4. Na subseção “Participantes”, linhas 43 e 44, sugere-se incluir “amostras de (secreção de) nasofaringe”

5. Na subseção “Participantes” como critério de exclusão os autores descreveram: “Foram excluídos indivíduos sem informações sobre status vacinal, data de hospitalização, ou variante do SARS-CoV-2, além daqueles com dados inconsistentes sobre o regime vacinal, como ausência de informações sobre data de vacinação, fabricante ou número de doses.”, porém, como o critério de determinação da variante foi epidemiológico faz-se necessário um maior esclarecimento do item destacado.

6. Na subseção “Fontes de dados e mensuração”. Sugere-se descrever os períodos considerados para cada variante de acordo com o critério adotado

7. Ocorreram casos de reinfecção e reinternação ? Como foram tratados na análise

8. Nos métodos, sugere-se a confecção de um diagrama de fluxo para melhor compreensão do leitos dos pacientes excluídos e das principais inconsistência encontradas na análise dos dados no banco. Este material poderia estar no texto ou como material suplementar.

Resultados:

9. Sugere-se esclarecer quanto aos sintomas analisados, estes foram os sintomas presentes no momento da internação?

10. Na tabela 2 e ao longo do texto, sugere-se rever análise quando comparado vacinado e não vacinado. Nesta tabela incluiu-se na coluna de vacinados os pacientes “parcialmente vacinados”. Há consenso na literatura que duas doses de qualquer vacina protegem contra doença grave e morte com maior eficácia.

Sugere-se rever essa análise e os resultados dela. Incluo aqui referencias.

https://doi.org/10.1016/S1473-3099(22)00345-0

https://doi.org/10.1002/jmv.29391

Discussão:

11. Na linha 15, sugere-se explicar o que os autores interpretam como “intervenções preventivas” neste contexto.

12. Sugere-se transferir o parágrafo de limitações para o penúltimo parágrafoda discussão e ainda reescrever exaltando os pontos fortes da pesquisa em seguida, algumas considerações: O tamanho da amostra é um ponto forte do estudo e a análise estatística escolhida pressupõe a melhor a escolha dos autores portanto “perdas” são esperadas em qualquer método. Sugerese reescrever com essa abordagem

13. Incluir na discussão abordagem sobre duração da imunidade induzida pela vacina, principalmente em idosos o que pode justificar um maior percentual de hospitalizados na onda Omicron.

14. No parágrafo: “ A vacinação completa demonstrou um efeito protetor substancial, com uma redução significativa no desconforto respiratório e em sintomas como febre e dispneia, conforme observado em um estudo multicêntrico realizado na Grécia entre junho de 2021 e fevereiro de 2022, com 265 pacientes

intubados.

